# Prenatal Exposure to Polychlorinated Biphenyls: A Neuropsychologic Analysis

**DOI:** 10.1289/ehp.11294

**Published:** 2008-08-14

**Authors:** Olivier Boucher, Gina Muckle, Célyne H. Bastien

**Affiliations:** 1 École de psychologie, Université Laval, Québec, Canada; 2 Unité de recherche en santé publique, Centre de recherche du CHUQ-CHUL, Québec, Canada; 3 Laboratoire de neurosciences comportementales humaines, Centre de recherche Université Laval-Robert Giffard, Québec, Canada

**Keywords:** attention, children, cognitive development, executive functions, memory, neuropsychologic profile, neurotoxicity, polychlorinated biphenyls, processing speed, review

## Abstract

**Objectives:**

A large body of literature documents the effects of prenatal exposure to polychlorinated biphenyls (PCBs) on cognitive development of children. Despite this fact, no integrative synthesis has been published yet to identify the cognitive functions that are particularly affected. Our aim is to review this literature in an attempt to identify the cognitive profile associated with prenatal PCB exposure.

**Data sources:**

Studies were identified by searching the PubMed database for articles published before June 2008. We reviewed data from nine prospective longitudinal birth cohorts for different aspects of cognition.

**Data extraction:**

Associations between indicators of prenatal PCB exposure and performance on cognitive tasks reported in the selected studies are summarized and classified as general cognitive abilities, verbal or visual–spatial skills, memory, attention, and executive functions.

**Data synthesis:**

The most consistent effects observed across studies are impaired executive functioning related to increased prenatal PCB exposure. Negative effects on processing speed, verbal abilities, and visual recognition memory are also reported by most studies. Converging results from different cohort studies in which exposure arises from different sources make it unlikely that co-exposure with another associated contaminant is responsible for the observed effects.

**Conclusion:**

Prenatal PCB exposure appears to be related to a relatively specific cognitive profile of impairments. Failure to assess functions that are specifically impaired may explain the absence of effects found in some studies. Our findings have implications in the selection of cognitive assessment methods in future studies.

Polychlorinated biphenyls (PCBs) are synthetic organochlorine compounds that were produced large scale from the early 1930s. Because of their unique physical and chemical properties, they were used largely in industry, notably as capacitor and transformer oils, hydraulic fluids, lubricating oils, and in plasticizers (Brinkman and De Kok 1980). Toward the end of the 1970s, their persistence in the environment was recognized, and their production was banned in most industrialized countries. More than 20 years after the prohibition of their production in North America, PCBs were still detectable in the environment because of inadvertent spills, careless disposal, their chemical stability, and resistance to biodegradation (Safe 1994).

Currently, one of the major nonaccidental sources of exposure to PCBs is fish consumption (Schwartz et al. 1983). Concentrations of PCBs are particularly high in predatory species, because their long half-life and strong lipophilicity cause their bioamplification in the food chain (Dewailly et al. 1993; Jensen 1987). PCB exposure in young children occurs first during prenatal development via placental transfer and then after birth from breast-feeding (Masuda et al. 1978; Patandin et al. 1997) and through contaminated food intake. Fetuses, infants, and children are especially vulnerable to environmental contaminants, more so than adults (Chance and Harmsen 1998), because of physiologic, anatomic, and behavioral features associated with development (e.g., fast brain growth and underdeveloped immune system).

The developmental neurotoxicity of PCBs was first recognized after large-scale poisoning episodes in Japan in 1968 and in Taiwan in 1979. The poisoning was associated with the intake of rice oil contaminated by PCB during production processes. Thousands of people displayed clinical signs of intoxication (Hsu et al. 1985). Children exposed *in utero*, whose mothers ingested contaminated rice oil prior to or during pregnancy, were the most affected. Many of them presented growth retardation, nail malformations, delays in cognitive development, and behavioral problems (Guo et al. 2004). Since these poisoning episodes, birth cohort studies to identify the effects of prenatal and perinatal exposure to background levels of PCBs from environmental sources on child cognition have been conducted in various countries. These studies usually reported associations between biologic markers of PCB exposure and child performance on various neuropsychological tests. Although most of these results have been summarized elsewhere (Schantz et al. 2003), no integrative synthesis aimed at portraying a profile of cognitive impairments from these results has been published yet. Earlier reports compared results by focusing on methodologic aspects such as exposure measurement. Albeit important and quite pertinent, these comparisons did not offer a comprehensive and detailed profile of specific assessed outcomes. Because of inconsistency among studies, some reviewers recently concluded that the effects of prenatal exposure to background levels of PCBs on child cognition are still not clearly established (Cicchetti et al. 2004; Ross 2004). Actually, it may well be that PCB exposure affects only certain cognitive functions rather than all aspects of cognitive functioning. Because neuropsychological assessment differs from one study to the other, part of the apparent inconsistency may result from the absence of theory-based comparisons between the cognitive outcomes of the different studies.

The main goal of the present literature review is to determine whether a distinctive cognitive profile associated with prenatal PCB exposure from environmental sources emerges from the studies conducted to date and if it does, to characterize it. Implications for future studies, notably in the selection of neuropsychological tests, will then be discussed.

## Methods

We searched the PubMed database (http://www.ncbi.nlm.nih.gov/sites/entrez?db=pubmed) for articles published up to June 2008. We included longitudinal prospective birth cohort studies with biologic markers of prenatal PCB exposure and indicators of cognition from infancy to childhood, and we report results pertaining to associations between PCB exposure and cognition. Associations between PCB exposure and cognition were considered statistically significant at *p* < 0.05. Significant results that were associated with a moderating variable were noted. The strengths and limitations of each study were considered in the cognitive neuropsychological analysis of effects. Inconsistencies between cohorts thought to be attributable to unique influences associated with a specific study are noted.

We included nine birth cohort studies on the effects of prenatal PCB exposure. Some particularities of these studies are summarized in Table 1. In all tables studies are presented in ascending order of severity of exposure. Because the laboratory procedures and types of specimens used for PCB exposure analysis varied across studies, the severity of exposure is estimated from the median PCB congener no. 153 (PCB-153) levels in maternal serum, based on the work of Longnecker and colleagues (2003). Selected studies were conducted between 1959 and 2008 in North America, Northern Europe, and Japan. The most frequent source of exposure was seafood consumption. In the following section we briefly describe these cohort studies in chronological order of publication.

### North Carolina

The North Carolina Breast Milk and Formula Project, initiated in 1978 in the Raleigh–Durham, North Carolina, USA, area was designed to evaluate the morbidity linked to PCB and dichloroethylene exposure in breast milk (Rogan et al. 1986a). PCB exposure was assumed to come from background levels in the general food supply rather than from specific dietary habits. Biologic samples from cord and maternal blood, breast milk, and placenta were obtained. More than 800 children were tested at 6 and 12 months of age. Approximately 700 were reassessed at 18 and 24 months of age and again between 3 and 5 years of age (Gladen et al. 1988; Gladen and Rogan 1991).

### Michigan

More than 8,000 women who gave birth in one of four western Michigan hospitals in 1980–1981 were interviewed after delivery. Among them, women who had ingested ≥ 26 lbs (about 11.8 kg) or more of Lake Michigan fish in the 6 preceding years were invited to participate. The initial sample consisted of 313 women. Seventy-seven percent reported moderate or large quantities of lake fish ingestion, and the other 23% were randomly selected from among those who did not report any lake fish consumption (Jacobson et al. 1986). Breast milk and cord and maternal blood samples were collected. Children were tested at 5 and 7 months of age, then again when they were 4 and 11 years old. At the last follow-up, the remaining sample size was 212.

### Faroe Islands

Faroe Islands residents are heavily exposed to PCBs and methylmercury (MeHg) because of their pilot whale blubber and meat intake and extensive fish consumption. In 1986–1987, mother–child dyads (*n* = 1,022) were recruited to take part in a study on the effects of exposure to these contaminants. PCB exposure was estimated for 435 cord tissue samples. More than 400 children with documented PCB exposure were tested at 7 years of age (Grandjean et al. 2001).

### Germany

Between 1993 and 1995, 171 mother–child dyads were recruited in three Düsseldorf hospitals. Cord blood and maternal milk samples were collected to determine PCB exposure. Despite high PCB levels found, the source of exposure is not documented. Children were tested at 7, 18, 30, and 42 months of age. At the 42-month follow-up, the remaining number of children was 91 (Walkowiak et al. 2001).

### Netherlands

The Netherlands cohort study was performed in the Groningen and the Rotterdam areas, where consumption of dairy products and meat were the main sources of PCB exposure (Patandin et al. 1999a). Half the women recruited breast-fed their child for at least 6 weeks; the other half preferred formula feeding. Cord blood, maternal blood, and breast milk samples were collected. Various dioxins and furans were analyzed in the biologic samples. Children were tested at 3, 7, 18, and 42 months of age, and again at 6.5 and 9 years. Of the 418 infants recruited at birth, data were available for 376 at 6.5 years of age (Vreugdenhil et al. 2002). The 9-year assessment included 83 children from Rotterdam (Vreugdenhil et al. 2004a).

### Oswego

The Oswego Newborn and Infant Development Project is a prospective longitudinal study conducted in the state of New York, USA. This study examined the behavioral effects of pre- and perinatal exposure to PCBs and other persistent organic contaminants (Stewart et al. 2000). The consumption of contaminated Lake Ontario fish was the source of PCB exposure. Cord blood, placenta, and breast milk samples were collected. MeHg, lead, dichloroethylene, hexachlorobenzene, and mirex levels were also documented. Child assessments were conducted at 6, 12, 38, and 54 months of age and again at 8 and 9.5 years of age. From the 309 initial participants, about 200 children were tested at the last follow-up phase (Stewart et al. 2008).

### Nunavik

Between November 1995 and March 2001, pregnant Inuit women from the three largest communities of the Hudson Bay coast (Northern Québec, Canada) were invited to participate in the Environmental Contaminants and Child Development Study. PCBs, other chlorinated pesticides, MeHg, lead, polyunsaturated fatty acids, and selenium levels were estimated from cord blood, maternal blood and hair, and breast milk samples (Muckle et al. 2001). More than 190 infants were tested at 6 and 12 months of age. At 66 months of age, between 1999 and 2001, an additional 110 children underwent neuromotor and neurophysiologic testing (Després et al. 2005; Saint-Amour et al. 2006). These children were initially part of the Cord Blood Monitoring Program, aimed at documenting levels of exposure to environmental contaminants in the Inuit population of Northern Québec (Muckle et al. 1998). The same contaminant and nutrient levels as those from the preceding study were analyzed from cord blood samples.

### Collaborative Perinatal Project

The Collaborative Perinatal Project (CPP) is a longitudinal study that followed the development of > 50,000 children born between 1959 and 1966 in the United States. About 42,000 pregnant women were recruited in 12 hospitals from 11 different cities across the country. Forty years later, data from this study were used to assess the effects of PCB exposure on child development (Daniels et al. 2003; Gray et al. 2005). More than 1,000 children were selected among those born singleton and for whom PCB levels in maternal blood were documented. Cognitive outcomes have been studied in relation to PCB exposure at 8 months and at 4 and 7 years of age. Analyses at 7 years of age included nearly 900 participants.

### Hokkaido

The Hokkaido Study on Environment and Children’s Health evaluated the effects of prenatal PCB and dioxins exposure on child development (Nakajima et al. 2006). Pregnant women were recruited between July 2002 and July 2004 in a Sapporo hospital in Japan. Maternal blood samples were analyzed for PCB exposure arising mainly from fish consumption. To date, 135 infants have been tested at 6 months of age.

## Results

### General cognitive abilities

#### Infant development

Table 2 summarizes the results for the six studies in which the Bayley Scales of Infant Development (Bayley 1969, 1993) was used. This examination provides two main scores: the Mental Development Index (MDI) and the Psychomotor Development Index (PDI). The items of the MDI comprise memory, habituation, problem solving, first number concepts, generalization, classification, vocalizations, language, and social skills (Bayley 1993). Only the German study reported a significant decrease in MDI score as a function of maternal breast milk levels of PCBs at 2 weeks postpartum, which may be considered an index of prenatal exposure, because it reflects maternal body burdens during pregnancy. The German and CPP cohorts were the most highly exposed cohorts. These results suggest that either the effects on MDI were observable only beyond a certain threshold of exposure or that MDI is not sensitive enough to detect subtle effects of PCBs. The PDI assesses control of the gross and fine muscle groups. The PDI is considered here because mental and motor functioning during infancy are likely to be interdependent (correlations between PDI and MDI range from 0.24 to 0.72 between 3 and 30 months of age) (Bayley 1993). Two studies of six—those from North Carolina and Germany—suggested persistent effects of PCB exposure on PDI score.

#### Child IQ

Results for IQ tests during childhood are presented in Table 3. Out of the nine birth cohort studies, six used IQ-type tests, and four of those reported a lower IQ score as a function of *in utero* exposure to PCBs. Thus, most studies suggest that prenatal exposure to PCBs affects the development of general cognitive competence. In the Netherlands, PCB exposure is related to lower score of general cognitive abilities in children born to younger mothers and parents with lower verbal IQ only, suggesting that parental characteristics and home environment might moderate the effects of the pollutant on cognitive development (Vreugdenhil et al. 2002). Furthermore, as depicted in Table 3, Verbal but not Performance IQ was affected in both the Michigan and the Oswego cohorts when assessed during late childhood. These results suggest long-lasting effects on verbal functions, whereas visual–spatial functions may be less sensitive to prenatal PCB exposure. This assumption must be explored in more detail using assessments of specific verbal and visual–spatial skills.

### Verbal versus visual–spatial skills

Table 4 summarizes the results obtained with tests of specific verbal functions. The grouping of results suggests that verbal functions particularly could be affected by PCB exposure. Vocabulary and verbal comprehension, both highly related to language skills, have been consistently associated with prenatal PCB exposure in all cohorts in which they were part of the study design. However, in the Faroe Islands, the association between exposure and vocabulary was no longer significant when MeHg was included in the statistical model. In the Michigan study, reading comprehension was affected by PCB exposure among 11-year-old children. Furthermore, higher-level processing of verbal abilities—verbal abstraction—has also been negatively related to prenatal PCBs at 11 years of age. Unfortunately, this finding was not replicated with the Faroese cohort.

Visual–spatial abilities include functions that are relatively independent from language and long-term memory. Associations between prenatal PCB exposure and specific visual–spatial functions are reported in four cohorts (Table 4). Measures of visuo-motor integration, mental rotation, and visual discrimination failed to show association with PCB exposure in the Michigan, Netherlands, and Faroese studies. On the other hand, a PCB effect on visuo-motor integration was reported in Oswego. However, this effect was observed at 3 years of age and was not replicated when children were tested again a year and half later. Altogether, these results support the assumption that verbal functions, notably vocabulary and verbal comprehension, are more likely to be affected by prenatal PCB exposure than visual–spatial functions.

### Memory

Associations between PCB exposure and various memory functions are shown in Table 5. In the Oswego and Michigan studies, effects of prenatal PCB exposure on visual recognition memory were observed during the first year of life with the Fagan Test of Infant Intelligence, but no effect was observed in the German study. Visual recognition memory was also affected at 4 years of age in the Michigan study. On the other hand, in the Netherlands, the delayed reproduction of the Rey Complex Figure was unaffected in children 9 years of age. The apparent contradictory results between the Michigan and the Netherlands studies might first be explained by the age difference. Second, both studies differed with regard to the functions assessed in both tests: The Sternberg Memory task (Michigan) has time pressure and requires sustained attention and working memory more manifestly than the Rey Complex Figure delayed copy (Netherlands). Moreover, the Sternberg Memory has a strong verbal component, whereas the Rey Complex Figure essentially relies on visual–spatial memory.

Episodic memory refers to contextualized memories of one’s own experiences and learning (Lezak et al. 2004; Mazeau 2003). For example, one can remember a day spent in Rome and the name of a person met on that day, as well as what was for dinner. Such contextualized memories were assessed in two of the nine selected studies: the Dutch and the Faroese. In the Dutch study, the most severely exposed children were not different from the least exposed on the Auditory-Verbal Learning Test, which contained both immediate and delayed recalls of verbal information. The children of the Faroese cohort were assessed with the California Verbal Learning Test, a test of list learning and verbal memory comparable to the Auditory-Verbal Learning Test. The absence of association between episodic memory and PCB exposure found in the Dutch study was corroborated in the Faroese cohort. Thus, episodic memory does not appear to be particularly affected by prenatal PCB exposure, despite the relatively high PCB concentrations observed in both cohorts.

Whereas episodic memory is contextualized, semantic memory refers to the general knowledge one possesses, which is disconnected from its encoding context (e.g., Rome is the capital of Italy) (Lezak et al. 2004). Although this type of memory was assessed only in the Michigan study, decreased performance as a function of prenatal PCB exposure was observed.

Finally, short-term memory has been assessed in four different PCB cohorts with different measures and at different ages. The Memory Scale index of the McCarthy Scales of Children’s Abilities (MSCA; McCarthy 1972) was administered in the North Carolina, the Netherlands, and the Michigan studies. This index integrates scores from four subtests, each assessing different processes related to short-term and working memory. The Memory Scale index decreased as a function of prenatal PCB exposure in the Netherlands and Michigan. The association found in Michigan was attributed to two specific subtests involving the auditory modality, whereas results on specific subtests were not reported for North Carolina and the Netherlands studies. PCB exposure did not affect learning a list of words in the Netherlands and the Faroese studies. The performance on numeric span measures was altered by PCB exposure at 4 and 11 years of age in Michigan, whereas no such alteration was found for the forward condition in the Faroe Islands at 7 years of age. Regrettably, the Michigan study did not report forward and backward spans separately. Because these two conditions are thought to reflect different processes [the forward condition is thought to assess short-term information retention and attention allocation, whereas the backward condition additionally involves the executive component of working memory (Lezak et al. 2004)], it is not possible to currently identify what caused the decrease in performance on the numeric span measures.

The analysis of the effects of prenatal exposure to PCBs on memory components reveals that consistent effects were found on visual recognition memory tasks with young children. Although effects on long-term verbal memory are not evident, some data suggest that short-term verbal memory is affected by this contaminant. However, the effects could also be the result of a decreased attention allocation or poorer executive functioning, both processes being involved during the completion of tasks.

### Attention and executive functions

Attention and executive functions (EFs) are unique aspects of human cognition, because they modulate all other cognitive functions. For example, attention allocation is required to learn new information, and sustained attention is necessary to perform optimally on a 2-hr neuropsychological battery testing. EFs are crucial for learning and retrieval strategies and are solicited to adapt to new testing situations. Because most cognitive functions are subordinated to attention and EFs, impairment of these functions might indirectly affect other components of cognitive functioning as well.

Table 6 summarizes the effects of prenatal PCB exposure on attentional functions. Several aspects of attention can be identified. First, selective or focused attention is the capacity to highlight the one or two important stimuli or ideas being dealt with while suppressing awareness of competing distracters (Lezak et al. 2004). In Michigan, performance on the Stroop Color-Word test, which requires the capacity to focus attention on the color of a word while ignoring the written name of the color, was not associated with prenatal PCB exposure in 11-year-old children. Nevertheless, in these children, PCB exposure was related to the number of omission errors on a digit cancellation test, which requires visual selective attention as much as visual scanning and processing speed.

Processing speed is a central aspect of attentional functioning. Decreased speed of information processing can have broad-ranging effects on attentional activities (Ponsford and Kinsella 1992; Spikman et al. 1996). Processing speed was assessed using reaction times during the completion of a cognitive task in the Michigan, Netherlands, and Faroe Islands cohorts. Slower reaction times as a function of prenatal PCB exposure were observed in most assessments. The effects of PCB exposure on processing speed were also tested with neurophysiologic measures. The Netherlands study used an auditory oddball paradigm to assess the P300 wave of the event-related potentials—a positive deflection of the electroencephalograph voltage occurring about 300 milliseconds after the detection of target stimuli—at the 9-year assessment (Vreugdenhil et al. 2004b). Latency of this component is thought to reflect stimulus evaluation duration independently of response-associated processes (Kutas et al. 1977; McCarthy and Donchin 1981). Higher prenatal PCB exposure was related to delayed P300 latency, which is consistent with behavioral data from other studies. The same result had previously been observed among Yu-Cheng children severely exposed to PCBs *in utero* who were assessed with a similar P300 protocol between 7 and 12 years of age (Chen and Hsu 1994). Those results suggest that speed of information processing is an aspect of attention particularly susceptible to impairment by prenatal PCB exposure.

Another aspect of attention is the capacity to maintain an attentional activity over a prolonged period of time, a concept referred to as sustained attention (Lezak et al. 2004). It is measured by observing the evolution in performance over time on a given task. Performance on the Continuous Performance Test, a well-known task of sustained attention, did not decline over time as a function of prenatal PCB exposure in the Oswego, Michigan, and Faroese cohorts. However, the fluctuation in reaction times of 9-year-old highly exposed Dutch children was greater than among less-exposed children on the Simple Reaction Time Test, a similar task. The Dutch study is thus the only study suggesting a PCB effect on sustained attention.

EFs are recruited for controlled action in new or complex situations, especially when well-learned, routine action schemas are no longer adequate to meet the demands of the task (Norman and Shallice 1986). In the current review, four aspects of EFs were considered: response inhibition, planning, set shifting, and the executive component of working memory. Associations between prenatal PCB exposure and these functions are summarized in Table 7. Poorer response inhibition has been consistently related to prenatal PCB exposure in both the Michigan and the Oswego cohorts. The significant effect reported in Oswego, one of the least-exposed cohorts, suggests that this aspect is likely to be very sensitive to PCB exposure. Effects on task planning were also observed in both cohorts that documented this EF (Netherlands and Michigan). However, in the Netherlands, planning effects were reported using the Tower of London test but not the copy of the Rey Complex Figure. These different tasks solicit different processes. For instance, the Tower of London test is more likely to require response inhibition (Miyake et al. 2000). Finally, set shifting and working memory, documented in 11-year-old children from the Michigan cohort, were affected by PCB exposure in non- breast-fed children. Altogether, these observations may suggest that EFs are particularly affected by prenatal PCB exposure. Replication of these results is needed before definite conclusions can be drawn.

### Sensory and motor functions

Performance on many neurobehavioral cognitive tasks can be affected by sensory impairments or motor difficulties. It thus appears important to make sure that the aforementioned PCB effects are not the indirect consequences of alterations in those modalities. This is the object of the final step of the present neuropsychological analysis. Studies documenting effects of PCB exposure on sensory functions are summarized in Table 8. Results suggest that it is possible that prenatal PCB exposure alters the auditory system in a very subtle manner. However, this effect, if present, is unlikely to be strong enough as to influence performance on neurobehavioral tasks requiring the auditory modality (e.g., language assessments and auditory spans).

Results from psychomotor development and motor functioning assessments are presented in Table 9. The global portrait of results suggests that motor functions during childhood are not particularly affected by prenatal PCB exposure. Thus, decreased performance on neurobehavioral tests requiring physical (e.g., hand, fingers) response (e.g., Continuous Performance Test, Tower of London) is more likely to be related to actual cognitive impairments rather than to specific motor dysfunction.

## Discussion

The present review suggests that prenatal exposure to PCBs is associated with a fairly specific profile of cognitive impairments in children. Among the cognitive functions assessed in the different studies, detrimental effects have been established more clearly for EFs. In many cases, negative effects have also been observed for speed of information processing, verbal abilities, and visual recognition memory. However, there is relatively little evidence of effects on visual–spatial abilities, episodic memory, and sustained attention. Effects appear to be independent of sensory and motor functions. More data are needed to document effects on other cognitive functions.

Most consistent results across studies suggest particular vulnerability of EFs to prenatal PCB exposure. Different studies have found that efficiency in planning, executive working memory, set shifting, and especially response inhibition decrease as a function of levels of PCBs. Because the prefrontal structures of the brain are thought to be of particular importance in these higher-order functions (Jurado and Rosselli 2007), one can hypothesize that the development of the prefrontal cortex is affected by PCB exposure. This hypothesis is in accordance with animal studies that showed disturbance in dopamine levels of the prefrontal cortex (Seegal et al. 2005) in rats exposed to PCB congeners *in utero*. However, other structures are also involved in the execution of controlled behaviors in humans (Pillon and Dubois 2005), and specific injury to prefrontal cortex alone is not likely to explain other particularities of the cognitive profile associated with prenatal PCB exposure (e.g., decreased processing speed; Vreugdenhil et al. 2004b). Thus, direct evidence of brain structural or functional alterations could help in relating prenatal PCB exposure to prefrontal cortex integrity (e.g., Cecil et al. 2008; Jonkman et al. 2007). EFs are sensitive to several disorders such as attention deficit and hyperactivity disorder, autism, and obsessive-compulsive disorder (Barkley 1997; Happé et al. 2006; Hughes et al. 1994; Olley et al. 2007), and to other neurotoxicants such as lead, marijuana, and cocaine (Canfield et al. 2004; Fried and Smith 2001; Mayes et al. 2005). This greater sensitivity to tasks designed to assess EFs may be related to their dependence on the integrity of multiple neural systems. Use of more focused tests would further our understanding of the specific end points responsible for the aforementioned deficits. In addition, failure to observe detrimental effects of PCB exposure on child cognition in the CPP, North Carolina, and Faroese cohorts might be related to the absence of specific EF assessments in those studies.

Although the effects of PCBs on the speed of information processing are strongly suggested by the present review, some questions still remain for another central aspect of attention—selective attention. First, only the Michigan study used specific tests of selective attention, and the results obtained were inconsistent. Second, the present literature review did not succeed in discriminating the relative contribution of attention allocation, working memory, and short-term memory to the effects observed on memory performance. For the moment, one can hypothesize that the documented effects on EFs are responsible for effects observed on numeric span subtests when the backward condition is used.

Other specific impairments caused by prenatal PCB exposure are highlighted by the distinction between verbal and visual-spatial functions; in most studies, the former are affected, whereas the latter are not. The distinction between verbal and visual–spatial functions is traditionally made in neuropsychology, notably to evaluate the differential integrity of each cerebral hemisphere (Samson 2005). However, some explanations other than specific effects on verbal hemisphere can be offered. For instance, verbal measures such as vocabulary and reading tests are more likely to be influenced by socioeconomic factors than are visual–spatial measures. If a given socioeconomic factor is related to sources of PCB exposure (e.g., fish products) in a given population, then specific associations between PCB exposure and lower scores on verbal tests could also reflect differences in socioeconomic conditions. Researchers should thus seek the verbal tasks that are less likely to be influenced by such confounding factors, such as measures of phonologic processes.

Although the present cognitive analysis highlights the effects of prenatal exposure to PCBs, inconsistencies between studies still persist. Many factors may explain these inconsistencies. First, it is possible that PCB exposure was not high enough to produce statistically significant effects in some cohorts. Strangely, though, differences in exposure severity do not appear to account for observed inconsistencies. Thus, studies reporting the larger number of significant effects on cognitive functions were not the most highly exposed cohorts. This is particularly true in the case of Oswego, which found consistent impairments on cognitive functions although it was among the least PCB-exposed cohorts.

It is also possible that co-exposure to other contaminants is accountable for inconsistencies across studies. Strong association between PCBs and another contaminant (e.g., MeHg) makes it less likely to observe independent relationships between PCBs and cognitive outcomes after documenting and statistically controlling for the other contaminant. This might explain the results observed in the Faroese, where statistical control for MeHg altered the associations between PCB exposure and cognitive outcomes (Grandjean et al. 2001). Exposure to another contaminant highly related to PCB levels might also be responsible for the observed cognitive effects if exposure to this contaminant is not documented and accounted for in statistical designs. However, if this were the case here, study results would diverge largely depending on the source of exposure in the population under study. In populations in which exposure arises from fish consumption (Michigan and Oswego), coexisting contaminants (e.g., MeHg) differ from those found in populations for whom exposure arises from dairy products (Netherlands). The consistent results obtained from those three cohorts suggest that the impairments associated with PCB exposure are more likely to be attributable to PCBs per se. In addition, statistical control for coexisting contaminants did not alter the relationships between PCB exposure and cognitive outcomes in Oswego (Stewart et al. 2003b, 2005, 2006, 2008).

Other explanations could be offered regarding inconsistent findings across cohort studies. Of particular importance is the presence of essential nutrients that may promote brain development in the sources of PCB exposure. This may be the case when exposure arises from fish products, which are also potential sources of omega-3 fatty acids (Costa 2007). If we assume that such nutrients have beneficial or protective effects on cognitive development (Koletzko et al. 2008), it is conceivable that the associations between prenatal PCB exposure and cognitive outcomes are stronger than what was actually found in fish-eating populations. In the Faroe Islands, attempts to control for the beneficial effects of fish consumption during pregnancy increased the effects of MeHg on cognition (Budtz-Jorgensen et al. 2007), but no such control was applied for PCBs. The Nunavik study, for which levels of nutrients are documented, will provide a unique opportunity in the near future to learn more about the relationships between nutrients and contaminants. Another explanation that cannot be ruled out is that different PCB mixtures with different congeners across studies result in different outcomes. As emphasized by Schantz et al. (2003), future studies will have to address this issue.

Age at assessment can also influence the outcomes of birth cohort studies. Although most cohort studies did not find significant effects of prenatal PCB exposure on the global mental development of infants, effects on global IQ tests were consistently observed during childhood in most studies (for example, effects on IQ were found in the Oswego, Netherlands, Michigan, and German studies). An explanation for such findings might be that infant mental development measures have weaker psychometric properties than childhood IQ tests in terms of reliability and validity, especially for predictive validity of later cognitive functioning (Bayley 1993; Wechsler 1991). In addition, because the maturation of certain brain areas, notably the prefrontal cortex, lasts until late adolescence (Segalowitz and Davies 2004), it is possible that some effects appear during development, while others disappear. PCBs were not related to global IQ at 4 years of age in the Michigan cohort but were at age 11 years (Jacobson et al. 1990; Jacobson and Jacobson 1996). Similar results were obtained in the Oswego study (Stewart et al. 2003b, 2008). These results outline the relevance of assessing cohorts until late childhood and adolescence.

In two cohort studies (Netherlands and Michigan), impairments associated with prenatal PCB were more salient in non-breast-fed and/or in more socioeconomically disadvantaged children. Thus, some of the findings reported in this literature review might hold only for more vulnerable subgroups. It is possible that optimal child stimulation can help compensate for subtle brain insults related to PCB exposure (Jacobson and Jacobson 2004), decreasing the likelihood of observing significant effects in more advantaged subgroups of the population. Failure to control for the appropriate vulnerability factors might also explain the negative findings from some studies. For instance, both studies that did not find effects on IQ (CPP and North Carolina) did not control for quality of parenting as measured by quality of home environment and parental verbal abilities. These variables were included in the analyses from the Michigan (11-year assessment), Oswego, Netherlands, and Germany cohort studies, all of which found significant effects. We cannot rule out the possibility that the CPP and North Carolina studies could have found PCB effects among more disadvantaged children in those variables. Particular attention to this issue should be given in future studies when reporting results. The CPP and North Carolina studies were the only two studies to use multiple testers at multiple sites. This could have caused increased variability in testing procedures, resulting in poorer reliability and reduced sensitivity of the measures.

The results of the present work can be used for the appropriate selection of neuropsychological tests in future studies of cohorts exposed to PCBs (or other similar organochlorine compounds). With the objective of corroborating the effects observed in birth cohort studies, we suggest that future studies systematically include the Fagan Test of Infant Intelligence (Fagan and McGrath 1981) during infancy and the Continuous Performance Test (Conners 1995; Letz and Baker 1988) from childhood. These tests have been found to be sensitive to the neurotoxic effects of PCBs on several occasions. Other tests of EFs, such as those from the Delis-Kaplan Executive Function System (Delis et al. 2001) or Go/No-go tasks, and tests requiring processing speed, like the Coding subtest of the Wechsler Intelligence Scales (Wechsler 1991), might also be sensitive to the presence of PCB effects. Further investigation of attentional and verbal/phonologic processes could also reveal pertinent information about the nature of the effects of prenatal PCB exposure. Subtests from neuropsychological assessment batteries used with children could be useful to this end. Among others, the Test of Everyday Attention for Children (TEA-Ch; Manly et al. 1999) battery is particularly well suited to disentangle processes required to perform attentional tasks, and the NEPSY (Korkman et al. 1998) battery has interesting subtests of phonologic processing.

We also suggest that if statistical power is high, analyses should be performed for every subtest rather than only for global indexes, especially if using test batteries such as the Wechsler Intelligence Scales for Children (WISC; Wechsler 1991), the MSCA (McCarthy 1972), and the Kaufman Assessment Battery for Children (K-ABC; Melchers and Preus 1994; Neutel et al. 1996). This could be accomplished with the existing data from the German and the Dutch cohorts, notably. Moreover, when using the Digit Span subtest (e.g., Wechsler 1991), analyses for both forward and backward conditions should be performed, because the processes involved for these conditions are qualitatively different. Even though such an approach increases the likelihood of making type 1 errors, it allows for appropriate comparison of results across studies and provides a better profile of the nature of the effects.

Finally, the use of complementary methods for neuropsychological assessment would be advantageous for the study of the neurotoxicity of PCBs and related compounds. The understanding of the brain mechanisms involved in the production of specific cognitive impairments would be improved with structural and functional brain imagery studies. Event-related potentials could increase our understanding about the specific aspects of cognitive function that are impaired by permitting the child’s performance to be evaluated for specific components arrayed across time. Such direct measures of brain activity are also less subject to bias by cultural, language, and socioeconomic factors.

In summary, this review supports the existence of specific, detrimental effects of prenatal exposure to environmental levels of PCBs on neuropsychological functioning in children. EF impairments were mainly outlined. Failure to assess this specific aspect of cognition may explain why some studies did not find significant relationships between prenatal PCB exposure and cognitive development. These effects, along with possible slower information processing and impairments in verbal abilities and visual memory, can be responsible for the IQ effects observed in most studies.

## Figures and Tables

**Table 1:** PCB exposure in the different selected birth cohort studies.

Cohort^a^	Initial no.	Birth year	Source of exposure	Median PCB-153 (ng/g fat)^b^	Reference
Hokkaido	135	2002–2004	Fish	23	Nakajima et al. 2006
Oswego	309	1991–1994	Fish	40	Darvill et al. 2000
North Carolina	859	1978–1982	Unspecific	80	Rogan et al. 1986b
Netherlands	418	1990–1992	Dairy products, meat	100	Patandin et al. 1999b
Nunavik (1st)	175	1995–1998	Fish, marine mammals	100	Muckle et al. 2001
Michigan	313	1980–1981	Fish	120	Schwartz et al. 1983
Germany	171	1993–1995	Not specified	140	Winneke et al. 1998
CPP	1,207	1959–1965	Unspecific	140	Daniels et al. 2003
Faroe Islands	435	1986–1987	Fish, marine mammals	450^c^	Grandjean et al. 2001

CPP, Collaborative Perinatal Project.

a The cohorts are presented in ascending order of median prenatal PCB-153 concentration.

b Estimated median of PCB-153 levels in maternal serum (Longnecker et al. 2003; Nakajima et al. 2006).

c This value was estimated from 173 maternal serum specimens collected in the second Faroese birth cohort (children born 8 years after the first Faroese cohort considered in the present article).

**Table 2:** Study results for mental and psychomotor development assessed with the Bayley Scales of Infant Development.

		Effect		
Cohort^a^	Age (months)	MDI	PDI	Reference
Hokkaido	6	–	–	Nakajima et al. 2006
North Carolina	6, 12, 18, 24	–, –, –, –	↓, ↓, –, ↓	Gladen et al. 1988; Rogan and Gladen 1991
Netherlands	3, 7, 18	–, –, –	↓, –, –	Koopman-Esseboom et al. 1996
Michigan	5	–	–	Jacobson et al. 1986
Germany	7, 18, 30	↓^b^, –^c^, ↓^b^	–, ↓^b^, ↓^b^	Winneke et al. 1998; Walkowiak et al. 2001
CPP	8	–	–	Daniels et al. 2003

Abbreviations: ↓, statistically significant decreased performance on the measure; –, absence of significant effect.

a Cohorts are presented in ascending order of median prenatal PCB exposure.

b These effects are in relation to the sum of PCBs 138, 153, and 180 in breast milk (2 weeks postpartum) rather then in cord plasma.

c A negative association between breast milk PCB levels and MDI score approaches statistical significance (*p* = 0.06)

**Table 3:** Study results for IQ-type tests.

Cohort^a^	Age (years)	Test	IQ_total_	Verbal IQ	Nonverbal/ Performance IQ	Reference
Oswego	3	MSCA	↓	–	↓	Stewart et al. 2003b
	4.5	MSCA	–	–	–	Stewart et al. 2003b
	9	WISC-III	↓	↓	–	Stewart et al. 2008
North Carolina	3–5	MSCA	–	–	–	Gladen and Rogan 1991
Netherlands	3.5	K-ABC	↓^b^			Patandin et al. 1999b
	6.5	MSCA	↓^c^			Vreugdenhil et al. 2002
Michigan	4	MSCA	–	↓	–	Jacobson et al. 1990
	11	WISC-R	↓	↓	–	Jacobson and Jacobson 1996
Germany	3.5	K-ABC	↓			Walkowiak et al. 2001
CPP	4	Stanford-Binet	–			Gray et al. 2005
	7	WISC	–	–	–	Gray et al. 2005

Abbreviations: ↓, statistically significant decreased performance on the measure; –, absence of significant effect. Tests: K-ABC, Kaufman Assessment Battery for Children (Overall Cognitive Scale; Melchers and Preus 1994; Neutel et al. 1996); MSCA, McCarthy Scales of Children’s Abilities (McCarthy 1972; Van Der Meulen and Smorkovsky 1985); Stanford-Binet (Broman et al. 1975); WISC, Wechsler Intelligence Scales for Children (Wechsler 1949, 1974, 1991).

a Cohorts are presented in ascending order of median prenatal PCB exposure.

b Decrease is significant among non-breast-fed children only.

c Decrease is significant in children born of younger mothers and parents with lower verbal IQ scores only.

**Table 4:** Study results for verbal and visual–spatial functions.

	Cohort^a^	Age (years)	Effect	Measurement	Reference
Verbal functions					
Vocabulary	Oswego	3	↓	MSCA Word Knowledge	Stewart et al. 2003b
		4.5	–	MSCA Word Knowledge	Stewart et al. 2003b
	Michigan	4	–	PPVT	Jacobson et al. 1990
		11	↓	WISC-R Vocabulary	Jacobson and Jacobson 1996
		11	↓	WRMT Word Comprehension	Jacobson and Jacobson 1996
	Faroe Islands	7	–b	BNT	Grandjean et al. 2001
Verbal comprehension	Netherlands	3.5	↓^c^	RLDS	Patandin et al. 1999b
Reading comprehension	Michigan	11	↓	WRMT Reading Comprehension	Jacobson and Jacobson 1996
Verbal abstraction	Michigan	11	↓	WISC-R Similitudes	Jacobson and Jacobson 1996
	Faroe Islands	7	–	WISC-R Similitudes	Grandjean et al. 2001
Visual–spatial functions					
Visuo-motor integration	Oswego	3	↓	MSCA Block Building	Stewart et al. 2003b
		4.5	–	MSCA Block Building	Stewart et al. 2003b
	Michigan	4	–	BTVMI	Jacobson et al. 1990
	Netherlands	9	–	Rey Complex Figure Test copy	Vreugdenhil et al. 2004a
	Faroe Islands	7	–	WISC Block Design	Grandjean et al. 2001
Mental rotation	Michigan	11	–	Mental Rotation	Jacobson and Jacobson 2003
Visual discrimination	Michigan	4	–	KMFF	Jacobson et al. 1992

Abbreviations: ↓, statistically significant decreased performance on the measure; –, absence of significant effect. Tests: BNT, Boston Naming Test (Kaplan et al. 1983); BTVMI, Beery Test of Visual Motor Integration (Beery 1967); KMFF, Kagan Matching Familiar Figures (Kagan 1965), Mental Rotation (Kail 1986); PPVT, Peabody Picture Vocabulary Test (Dunn and Dunn 1981); Rey Complex Figure Test copy (Rey 1941); RLDS, Reynell Language Development Scales (Van Eldik et al. 1995); WRMT, Woodcock Reading Mastery Tests (Woodcock 1987).

a Cohorts are presented in ascending order of median prenatal PCB exposure.

b Decreased performance as a function of prenatal PCB exposure was significant before controlling for MeHg.

c Effect is significant among non-breast-fed children only.

**Table 5:** Study results on verbal and visual memory.

		Age				
Assessed construct	Cohort^a^	Months	Years	Effect	Measurement	Reference
Visual recognition	Oswego	6, 12		↓	FTII	Darvill et al. 2000
	Michigan	7		↓	FTII	Jacobson et al. 1985
	Michigan		4	↓	Sternberg Memory^b^	Jacobson et al. 1992
	Germany	7		–	FTII	Winneke et al. 1998
Visual reproduction	Netherlands		9	–	Rey Complex Figure Test recall	Vreugdenhil et al. 2004a
Episodic memory	Netherlands		9	–	AVLT long delay recall	Vreugdenhil et al. 2004a
	Faroe Islands		7	–	CVLT long delay recall	Grandjean et al. 2001
Semantic memory	Michigan		11	↓	WISC Information	Jacobson and Jacobson 1996
Short-term memory	North Carolina		3–5	–	MSCA Memory Scale^c^	Gladen and Rogan 1991
	Netherlands		6.5	↓^d^	MSCA Memory Scale^c^	Vreugdenhil et al. 2002
			9	–	AVLT short delay recall	Vreugdenhil et al. 2004a
	Michigan		4	↓	MSCA Memory Scale	Jacobson et al. 1990
			11	↓^e^	WISC-R Digit Span	Jacobson and Jacobson 2003
			11	–	Corsi Spatial Span	Jacobson and Jacobson 2003
			11	↓	Sternberg Memory	Jacobson and Jacobson 2003
	Faroe Islands		7	–	WISC-R Digit Span Forward	Grandjean et al. 2001
			7	–	CVLT short delay recall	Grandjean et al. 2001

Abbreviations: ↓, statistically significant decreased performance on the measure; –, absence of significant effect. Tests: AVLT, Auditory-Verbal Learning Test (Kalverboer and Deelman 1964); Corsi Spatial Span (Corsi 1972); CVLT, California Verbal Learning Test (Delis et al. 1994); FTII, Fagan Test of Infant Intelligence (Fagan and McGrath 1981); Sternberg Memory [adaptation from Sternberg (1969)].

a Cohorts are presented in ascending order of median prenatal PCB exposure.

b This adaptation of the Sternberg Memory Test emphasizes visual recognition memory rather than working memory.

c Authors do not report associations with the different subtests of the Memory Scale.

d Effect significant in children of younger mothers and parents with lower verbal IQ scores only.

e Effect significant in non-breast-fed children only.

**Table 6:** Results for attention.

Assessed construct	Cohort^a^	Age (years)	Effect	Measurement	Reference
Selective attention	Michigan	11	↓	Digit Cancellation omission errors	Jacobson and Jacobson 2003
		11	–	Stroop Color-Word completion time	Jacobson and Jacobson 2003
Processing speed	Michigan	4	↓	KMFF reaction time	Jacobson et al. 1992
		4, 11	–	CPT reaction time	Jacobson and Jacobson 2003
		11	↓	Mental Rotation reaction time	Jacobson and Jacobson 2003
	Netherlands	9	↓	SRTT reaction time	Vreugdenhil et al. 2004a
		9	↓	P300 latency	Vreugdenhil et al. 2004b
	Faroe Islands	7	–b	CPT reaction time	Grandjean et al. 2001
Sustained attention	Oswego	4.5	–	CPT	Stewart et al. 2003a
		8, 9.5	–	CPT	Stewart et al. 2005
	Netherlands	9	↓	SRTT reaction time variations	Vreugdenhil et al. 2004a
	Michigan	4, 11	–	CPT	Jacobson and Jacobson 2003
	Faroe Islands	7	–	CPT	Grandjean et al. 2001

Abbreviations: ↓, statistically significant decreased performance on the measure; –, absence of significant effect. Tests: CPT, Continuous Performance Test (Letz and Baker 1988; Rosvold et al. 1956); SRTT, Simple Reaction Time Test (Letz 1994).

a Cohorts are presented in ascending order of mean prenatal PCB exposure.

b Decreased performance as a function of prenatal PCB exposure was significant before documenting relative effect of MeHg.

**Table 7:** Study results on EF.

EF	Cohort^a^	Age (years)	Effect	Measurement	Reference
Response inhibition	Oswego	4.5	↓	CPT commission errors	Stewart et al. 2003a
		8, 9.5	↓	CPT commission errors	Stewart et al. 2005
		9.5	↓	DRL inter-response times	Stewart et al. 2006
	Michigan	4	–	CPT commission errors	Jacobson and Jacobson 2003
		4	↓	Sternberg Memory errors of commission	Jacobson and Jacobson 2003
		11	↓^b^	CPT commission errors	Jacobson and Jacobson 2003
Planning	Netherlands	9	↓	Tower of London	Vreugdenhil et al. 2004a
		9	–	Rey Complex Figure Test copy strategy	Vreugdenhil et al. 2004a
	Michigan	11	↓	WISC-R Labyrinth	Jacobson and Jacobson 1996
Set shifting	Michigan	11	↓^b^	WCST perseverative errors	Jacobson and Jacobson 2003
Working memory	Michigan	11	↓^b^	WISC-R Arithmetic	Jacobson and Jacobson 2003

Abbreviations: ↓, statistically significant decreased performance on the measure; –, absence of significant effect. Tests: DRL, Differential reinforcement of low rates schedules [based on Sagvolden et al. (1998)]; Tower of London (Shallice 1982); WCST, Wisconsin Card Sorting Test (Grant and Berg 1948); WISC, Wechsler Intelligence Scale for Children (Wechsler 1991).

a Cohorts are presented in ascending order of median prenatal PCB exposure.

b Decrease is significant only in non-breast-fed children.

**Table 8:** Study results on auditory and visual functioning.

Assessment	Cohort	Age (years)	Effect	Reference
Hearing	CPP	8	–	Longnecker et al. 2004
	Faroe Islands	7	↓^a^	Grandjean et al. 2001
BAEPs	Faroe Islands	7	–b	Grandjean et al. 2001
Visual contrast sensitivity	Faroe Islands	7	–	Grandjean et al. 2001
VEPs	Nunavik	5	–c	Saint-Amour et al. 2006
	Faroe Islands	7	–	Grandjean et al. 2001

Abbreviations: ↓, statistically significant decreased performance on the measure; –, absence of significant effect; BAEPs, brainstem auditory evoked potentials; VEPs, visual evoked potentials.

a Significant increase of auditory threshold in left ear only for sounds of 250 and 12,000 Hz.

b Delayed latency of wave V at 20 Hz was significant before controlling for MeHg.

c P100 and N150 waves latencies were significantly delayed, and N75 to P100 and P100 to N150 amplitudes were significantly reduced as a function of child’s PCB-153 levels in blood at 5 years of age.

**Table 9:** Effects of prenatal exposure to PCBs on motor function.

Motor assessment	Cohort	Age (years)	Effect	Measurement	Reference
Global motor	Netherlands	6.5	↓^a^	MSCA Motor Scale	Vreugdenhil et al. 2002
	Michigan	4	–	MSCA Motor Scale	Jacobson et al. 1990
Gross motor	Nunavik	5	–	Huttenlocher gross motor tasks	Després et al. 2005
Fine neuromotor	Nunavik	5	–	Catsys System hand tremor	Després et al. 2005
		5	–	Catsys System postural sway	Després et al. 2005
		5	–	Catsys System reaction time	Després et al. 2005
		5	–	Rapid pointing movements	Després et al. 2005
		5	–	Rapid alternative arm movements	Després et al. 2005
	Faroe Islands	7	–	NES-2 Finger Tapping	Grandjean et al. 2001
		7	–	NES-2 Hand-eye Coordination	Grandjean et al. 2001

Abbreviations: ↓, statistically significant decreased performance on the measure; –, absence of significant effect. Tests: Catsys system (Danish Product Development 2000); Huttenlocher gross motor tasks (Huttenlocher et al. 1990); NES-2, Neurobehavioral Evaluation System (Letz and Baker 1988).

a Significant in children with poorer environmental conditions only.
